# Acupuncture in persons with an increased stress level—Results from a randomized-controlled pilot trial

**DOI:** 10.1371/journal.pone.0236004

**Published:** 2020-07-23

**Authors:** Beate Wild, Judith Brenner, Stefanie Joos, Yvonne Samstag, Magdalena Buckert, Jan Valentini

**Affiliations:** 1 Department of General Internal Medicine and Psychosomatics, Medical University Hospital Heidelberg, Heidelberg, Germany; 2 Institute for General Practice and Interprofessional Care, Medical University Hospital Tübingen, Tübingen, Germany; 3 Institute of Immunology, Section Molecular Immunology, Heidelberg University, Heidelberg, Germany; Weill Cornell Medical College Qatar, QATAR

## Abstract

**Background:**

In today’s Western societies a high percentage of people experience increased or chronic stress. Acupuncture could serve as treatment for persons affected adversely by the increased stress.

**Methods:**

The AkuRest study was a two-centre randomized controlled pilot study in adult persons with increased stress levels. Participants were randomly allocated to one of three groups: verum acupuncture treatment, sham acupuncture, and a waiting control group. The feasibility of the study was assessed. In addition, effects on stress level (measured by the Perceived Stress Questionnaire (PSQ-20)) and other variables were assessed at the end of treatment and a 3-month follow-up.

**Results:**

Altogether, N = 70 persons were included in the study. At the end of the treatment 15.7% were lost to follow-up. The adherence to the protocol was good: 82.9% of the participants completed 100% of their treatment. The stress level of the participants was high at baseline (mean PSQ-20 score 75.5, SD = 8.2). Effect sizes (ES) at T1 showed that verum and sham acupuncture were superior to the waiting condition in reducing stress (ES (verum) = -1.39, 95%-CI = [-2.11; -0.67]: ES (sham) = -1.12, CI = [-1.78;-0.44]). At follow-up, effect sizes were in favour of the verum group (as compared to sham). However, confidence intervals and t-tests showed that these differences were not significant.

**Conclusion:**

The pilot study demonstrated the feasibility of the acupuncture RCT in persons with increased stress levels. Estimated parameters can be used to design a larger RCT to prove the–here indicated—efficacy of verum acupuncture to decrease stress.

**Trial registration number:**

ISRCTN15259166.

## Introduction

In Western societies today a high percentage of people are experiencing increased or chronic stress. The demands of work and the private lives of adults are often challenging and require considerable effort to sustain [1]. It is well-known that chronic stress is a risk factor (or intensifier) for a variety of physical disorders or illnesses [2, 3]. Chronic stress has been demonstrated to increase cardiovascular risks, musculoskeletal disorders, and mental disorders such as depression [4, 5]. There is a large body of literature examining increased stress levels of the various professions [6, 7]. In many professions, the prevalence of burnout as a possible consequence of chronic stress is increasing [8–10]. In light of a highly stressed and unbalanced working society the importance of short and effective treatment to reduce stress is undeniable.

Acupuncture, a part of the Traditional Chinese Medicine (TCM), has long been used as a treatment for stress-related disorders. It is a more than a 2000 year old empirical medicine whose efficacy for the treatment of various disorders such as low back pain and knee osteoarthritis has been confirmed [11–14]. In persons with chronic stress the balance between the sympathetic and parasympathetic nervous system is disturbed [5]. Basic research regarding the central effects of acupuncture showed that acupuncture is able to balance the sympathetic (activating) and parasympathetic (regenerating) parts of the vegetative nervous system [15, 16]. Chen and Liu found that acupuncture can indeed improve the symptoms of adrenal insufficiency as well as influence the regulation and regeneration of the sexual and adrenal glands. Beissner et al. [17] demonstrated that the needling sensation–the DeQi—activated the central autonomic network of the hypothalamus, periaquaeductal grey, and medulla. As shown by Mehta et al. [18], a series of acupuncture treatments over a longer period of time resulted in an increased activity of parasympathetic functions. These effects are consistent with the hypothesis that TCM acupuncture causes autonomic remodeling, most likely by reducing the sympathetic activity and increasing the parasympathetic.

To date, a few studies have shown that acupuncture may serve as treatment for persons with increased stress [19, 20]. However, well-designed randomized-controlled (RCT) studies demonstrating the efficacy of acupuncture in chronic stress are still lacking. The present study was a randomized-controlled pilot trial for adult persons with increased stress levels. The aim of the study was to determine the feasibility of implementing a verum acupuncture intervention (VA) versus a sham acupuncture treatment and as well as a wait list control condition (WLC). The second aim was the estimation of possible effect sizes at the end of treatment.

## Materials and methods

### Study design and participants

AkuRest was a randomized controlled pilot trial of adult persons with increased stress levels. The study was conducted at the University Hospital of Heidelberg and the University Hospital of Tübingen. Between September, 2017 and August, 2018 we included 70 participants. Inclusion criteria were a Perceived Stress Questionnaire (PSQ) Score ≥ 60, age ≥ 18 and written informed consent. Exclusion criteria were suicidal ideation, psychiatric disorder, needle phobia, and insufficient knowledge of the German language. We are aware that there are more factors that could interfere with stress levels. However, inclusion and exclusion criteria for the study were reduced to a minimum because we wished to show that acupuncture treatment would be feasible in general for the group of persons with high stress levels.

The trial was approved by the independent ethics committees of the universities Heidelberg (February, 2017) and Tübingen (July, 2017). It was registered in advance at http://www.isrctn.com/, number ISRCTN15259166. The authors confirm that all ongoing and related trials for this intervention are registered.

### Sample size

The sample size calculation was based on the confidence interval approach from Cocks & Torgersen [21] and the assumption that an effect size of 0.4 (regarding the stress level at the end of treatment) between the verum and sham acupuncture would be clinically relevant. A sample size of n = 42 would produce an upper limit of a one-sided 90%-interval that excludes 0.4, assuming that the treatment estimate of the pilot study was zero or less [21]. However, with a loss-to-follow up rate of about 16% a sample size of n = 50 (= 2 x 25) participants was needed for the verum and the sham acupuncture group. An additional n = 25 participants were added to the waiting control group, resulting in n = 75 participants for the pilot trial.

### Randomization and masking

Participants were randomized to one of three groups. Randomization was performed by using a stratified permuted block design with blocks of size n = 25 and centre as stratification variable. Randomization was conducted by using the randomization software “RANDI2”[22]. The randomization program was applied by an independent assistant at the University Hospital Heidelberg; data analysts were blinded to the assignment. Participants who were randomized to either the verum or the sham acupuncture group were blinded to treatment assignments that is, they did not know whether they were receiving verum or sham acupuncture.

### Treatments

The intervention is reported according to the STRICTA guidelines [23]. The acupuncture treatment consisted of 10 sessions. Each session was from 20–30 minutes in length) and consisted of approximately a 5 minutes consultation, 5 minutes needle insertion, and 20 minutes needle retention time. Intervals between treatments varied from 3 to 7 days. However, according to feasibility and the situation of the participants (e.g. holidays or illness) longer intervals between treatments were allowed. Acupuncture was performed by two medical doctors with an additional specialization in acupuncture provided by the medical association of Baden-Wuerttemberg, Germany (a minimum 200 teaching units required). Each acupuncturist had more then 10 years of experience in the field of acupuncture and traditional Chinese medicine. For all acupuncture sessions, sterile, silicone coated, single-use filiform acupuncture needles, with a length of 25 mm and a diameter of 0.25 mm each, were used. The manufacturer brand was not defined. For the verum acupuncture a semi-standardized protocol was defined in a consensus process between the acupuncturist and the study investigators, according to literature research, clinical expertise of the acupuncturists and results from previous studies [19]. For both groups traditional Chinese medicine acupuncture style was used. The acupuncture treatments were performed in an outpatient clinic of the participating university clinics. Based on the information given by Brinkhaus et al. [24] in a previous trial patients were informed about the types of acupuncture interventions in the study as follows: ‘‘In this study, different types of acupuncture will be compared. One type is performed according to the principles of traditional Chinese medicine; the other type does not follow these principles, but has also been associated with positive outcomes in clinical studies”.

To ensure the adherence to the standardized protocol both acupuncturists were continuously in contact with the study coordinators in Heidelberg and Tübingen. However, there were no site visits to ensure that they were following the standardized procedure.

For verum acupuncture three points were fixed (*Shenmen* HT7 und *Taixi* KI3 needled bilaterally, and *Neiguan* PC6 unilaterally) and maintained throughout the 10 sessions. These points were chosen according Chinese medicine theory in order to address the diagnostic patterns of Shaoyin and Jueyin syndromes which are frequently presented in persons with increased stress levels. Furthermore the selection of these acupuncture points is supported by preliminary evidence describing a potential influence on the regulation of stress [19, 25, 26]. In addition to the three fixed acupuncture points up to four points could be individually selected by the acupuncturist (with a maximum of 7 acupuncture points and 12 needles in total). The individual points could be chosen and altered during the course of treatment by the acupuncturist in accordance with the participants’ main clinical symptoms. In the verum acupuncture treatment depth of insertion was point-specific with the aim of eliciting a De Qi; after achieving De Qi no further needle stimulation techniques were applied. No other interventions (e.g. moxibustion, cupping, life style advice) were administered to the acupuncture groups.

The active control condition for the verum acupuncture was a sham acupuncture treatment. For the sham acupuncture, 4 to 6 standardized points which were not located on acupuncture meridians were chosen for acupuncture in a consensus process between the acupuncturist and the study investigators (non-acupuncture points). These points were needled only superficially, that is, without eliciting the so-called De Qi sensation; the control acupuncture points could be changed individually during the course of treatment similar to the verum acupuncture (maximum 12 needles). Further needling details and treatment regimen did not differ from the verum acupuncture treatment.

Participants in the waiting control group received no acupuncture treatments over the course of three months, after which they were offered a verum acupuncture treatment. We chose this procedure for the control group to enhance the participant’s motivation for the study and to prevent premature drop-outs.

### Outcome measures

For all participants, diagnostic assessments were done at T0 (baseline, after inclusion and before randomization), T1 (at the end of treatment or waiting time), and T2 (three months follow-up for the verum and sham groups; end of treatment for the waiting list group). At the three time points participants completed a study questionnaire. In addition, heart rate variability was recorded and blood and urine samples were taken.

The main aim of the pilot study was to assess the feasibility and acceptability of the study protocol. Feasibility and acceptability were assessed by means of the recruitment rate (proportion of eligible persons and included persons), the study dropout rate (defined as proportion of randomized patients to patients with incomplete data at T1 or T2), and completer rates.

In addition, effect sizes for verum and sham acupuncture were estimated at end of treatment and at follow-up regarding the reduction of stress levels and possible improvement in further psychosocial or endocrinologic parameters.

The main instrument for measuring stress was the Perceived Stress Questionnaire (PSQ-20) that aims to measure the subjective perception, appraisal, and processing of stressors [27, 28]. The questions are non-specific and can be interpreted to various situations (e.g. “You feel under pressure from deadlines”) [29]. The questionnaire queries stressful feelings and experiences over the course of the last month and shows a dimensional structure of four factors: “worries, tension, joy, and demands” [30]. On a scale from 1 (“almost never”) to 4 (“usually”), participants indicate how frequently they experience stress-related feelings or situations. A total score is obtained by a specific algorithm that transforms the raw score to a scale that ranges from 0–100. A PSQ-score ≥ 60 indicates a high stress level. The PSQ-20 has been used in various settings and has shown a sensitivity to change [28]. In addition to the PSQ-20, we applied the stress module of the Patient Health Questionnaire [31].

Depressive symptoms were assessed using the 9-item depression module of the Patient Health Questionnaire (PHQ-9) [32]. The PHQ-9 asks for cognitive, affective, and somatic depression symptoms; each item corresponds to one of the nine DSM-IV diagnostic A-criteria for a major depressive disorder; each item ranges from 0 to 3.

Somatic symptom severity of the participants was assessed using 13 items from the PHQ-15 questionnaire that is comprised of 15 somatic symptoms (stomach pain, back pain, etc.) each symptom is scored from 0 to 2 [33]

The Generalized Anxiety Disorder Scale (GAD-7) was applied to assess the symptom severity of GAD; the total score of the GAD-7 ranges from 0 to 21. The German version of the GAD-7 proved to be a reliable and valid instrument for screening for GAD [34, 35].

The Measure Yourself Medical Outcome Profile (MYMOP) is a four-item instrument that allows patients to name up to two symptoms that are most concerning to them, and to assess the change of these symptoms over time [36]. A profile score can be calculated by using the mean of the four ratings for the most important self-reported symptom. A higher profile score reflects a higher symptom burden.

Health-related quality of life was measured by the visual analogue scale (VAS) of the EQ-5D. The EQ-VAS records the respondent's self-rated health on a vertical VAS ranging from 0 to 100 where the endpoints are labelled “worst imaginable health state” (0) and”best imaginable health state” (100) [37]

For the determination of cortisone and cortisol levels, the first morning urine of the participants was collected and frozen until further analysis. The content of cortisone and cortisol in these samples was determined by Ultra Performance Liquid Chromatography (Waters ACQUITY UPLC) followed by tandem-mass spectrometry (LCMSMS, Ganzimmun).

### Statistical analysis

The feasibility of the study was measured descriptively using percentages. Mean values and standard deviations per group were calculated for the various variables and measurement time points. Repeated measurement ANOVAs were run to compare the three groups at T0 and T1. According to the Bonferroni-Holm-Shaffer method we applied pairwise t-tests (for T1 values) whenever the global F-test showed a p-value < 0.05. For T2, we only compared the two verum and sham acupuncture groups due to the fact that between T1 and T2 the waiting control group received verum acupuncture and could thus not serve as a control group at T2. For T2 we applied t-tests for change scores to analyse the differences between the verum and sham groups.

In addition, Cohen’s effect sizes for T1 were estimated together with their confidence intervals, to illustrate the differences between groups. For T2, effect sizes were calculated for the comparison between the verum and sham treatments only- as the waiting control group could not serve as a control group at T2. Effect sizes were calculated only for participants with complete data.

To explore the heterogeneity across the two study centres additional ANOVAs for T1-T0 and T2-T0 change scores (PSQ-20, PHQ stress, depressive symptoms, and anxiety) were conducted with treatment arm (verum vs. sham), centre, and treatment x centre as factors. All analyses were done by using the statistics software SAS, version 9.4.

## Results

### Participant characteristics

**Fig 1:**
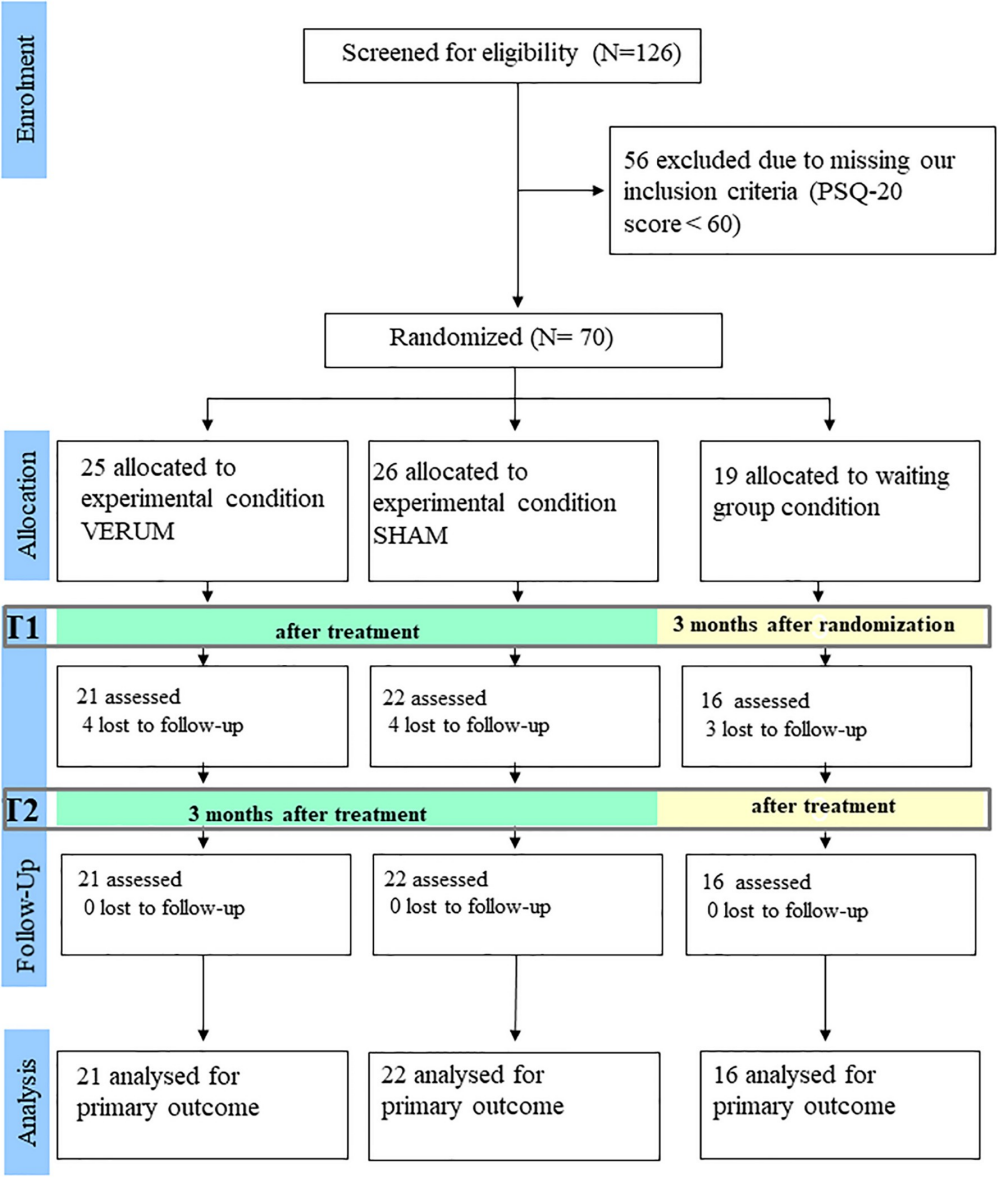
Trial flow of the participants.

Following an advertisement and distribution of fliers for the study N = 126 persons contacted the study centers in Heidelberg and Tübingen who were interested in participating in the trial. Of these 126 persons n = 70 (55.6%) met the eligibility criteria and were included in the study. At the end of treatment (or waiting time) 11 participants (15.7%) were lost to follow-up. The trial flow of the participants is shown in Fig 1.

Table 1 shows the baseline characteristics of the 70 study participants included in the pilot trial.

**Table 1:** Baseline characteristics of the study participants.

	Participants Heidelberg	Participants Tübingen	Participants in both study centers
	(n = 41)	(n = 29)	(n = 70)
**Demographic characteristics**			
Age (MW ± Std)	43.4 ± 13.2	52.1 ± 9.6	47.0 ± 12.5
Sex (n; %)			
*male*	12 (29.3)	7 (24.1)	19 (27.1)
*female*	29 (70.7)	22 (75.9)	51 (72.9)
Marital status (n; %)			
*single*	19 (46.3)	7 (24.1)	26 (37.1)
*married*	17 (41.5)	16 (55.2)	33 (47.1)
*divorced / widowed*	5 (12.2)	6 (20.7)	11 (15.7)
Education (years) (n; %)			
*≤ 9*	3 (7.3)	6 (20.7)	9 (12.9)
*10–11*	7 (17.1)	5 (17.2)	12 (17.1)
*≥ 12*	29 (70.7)	18 (62.1)	47 (67.1)
*Other*	2 (4.9)	0 (0.0)	2 (2.9)
**Clinical characteristics**			
PSQ-20 Screening Score (MW ± Std)	73.9 ± 8.5	70.9 ± 7.9	72.6 ± 8.3
PSQ-20 Baseline Score (MW ± Std)	74.3 ± 7.8	77.3 ± 8.6	75.5 ± 8.2
EQ-5D (Visual analogue scale) (MW ± Std)	65.2 ± 20.9	64.2 ± 15.0	64.8 ± 18.6
Depression severity (PHQ-9) (MW ± Std)	10.8 ± 4.5	12.0 ± 4.8	11.3 ± 4.6
Anxiety severity (GAD-7) (MW ± Std)	9.8 ± 4.3	11.5 ± 4.8	10.5 ± 4.5

PSQ-20 = Perceived Stress Questionnaire; GAD-7 = Generalized Anxiety Disorder Scale; PHQ-9 = Patient Health Questionnaire

The overall mean PSQ-20 score of 75.5 at baseline reflects the high stress level of the included participants. Participants also showed high mean values in depression and anxiety severity at baseline.

Overall, the participants showed a high compliance with the treatment protocol. N = 58 (82.9%) completed 100% (= 10 acupuncture sessions); 3 persons completed at least 80% of their treatment while N = 7 participants did not start the acupuncture sessions due to various reasons such as an accident or inaccessibility.

Specifically, 16 participants from the waiting list group received verum acupuncture between T1 and T2 (84.2%). Of these, 15 completed 100% of their treatment.

No serious adverse events attributable to trial participation were recorded.

### Effect sizes of stress related variables

Table 2 illustrates the mean values for the PSQ-20 and additional stress-related variables for the three groups at the various measurement time points.

**Table 2:** Mean values for the PSQ-20 and further stress-related variables at three time points.

	(v) Verum acupuncture T0: n = 25 T1: n = 21 T2: n = 21 mean (s.d.)	(s) Sham acupuncture T0: n = 26 T1: n = 22 T2: n = 22 mean (s.d.)	(w) Waiting control group T0: n = 19 T1: n = 16 T2: n = 16 mean (s.d.)	Contrast for T1-T0 scores (v)–(s) t-value (df) p-value	Contrast for T1-T0 scores (v)–(w) t-value (df) p-value	Contrast for T1-T0 scores (s)–(w) t-value (df) p-value
PSQ-20 (T0)	75.2 (8.2)	76.7 (9.2)	74.3 (6.9)	-1.41 (41)	-4.1 (35)	-3.4 (38)
PSQ-20 (T1)	46.5 (19.5)	55.2 (13.2)	68.0 (11.4)	0.17	0.0002	0.002
PSQ-20 (T2)	51.6 (21.5)	58.2 (13.3)	46.7 (12.7)			
PHQ stress module (T0)	8.5 (3.8)	8.7 (4.6)	7.7 (3.6)	-0.20 (41)	-2.34 (35)	-1.6 (38)
PHQ stress module (T1)	5.3 (3.6)	6.2 (3.9)	6.6 (3.4)	0.84	0.03	0.11
PHQ stress module (T2)	5.8 (4.)	7.0 (2.9)	4.3 (2.3)			
Depression severity (T0)	11.6 (4.4)	11.8 (4.4)	10.2 (5.3)	-0.19 (41)	-3.05 (35)	-2.93 (38)
Depression severity (T1)	5.6 (2.9)	6.0 (3.7)	8.1 (4.3)	0.85	0.004	0.006
Depression severity (T2)	6.5 (3.3)	7.0 (3.0)	4.8 (2.3)			
Generalized anxiety (T0)	11.6 (4.9)	10.0 (4.2)	9.6 (4.4)	-1.46 (41)	-3.08 (34)	-1.99 (37)
Generalized anxiety (T1)	5.7 (3.1)	5.2 (3.6)	8.4 (4.4)	0.15	0.004	0.05
Generalized anxiety (T2)	5.9 (4.1)	5.9 (3.7)	4.8 (2.6)			
Somatic complaints (T0)	6.4 (4.2)	8.6 (4.9)	6.5 (3.1)	0.11 (41)	-3.08 (35)	-2.75 (38)
Somatic complaints (T1)	3.6 (3.0)	5.7 (3.3)	6.9 (3.4)	0.92	0.004	0.009
Somatic complaints (T2)	4.7 (3.9)	5.9 (3.2)	5.1 (2.9)			
Quality of life (T0)	65.3 (18.3)	62.2 (21.1)	67.6 (15.5)	-0.00 (40)	2.28 (34)	2.44 (38)
Quality of life (T1)	79.6 (12.9)	73.7 (12.8)	66.1 (15.0)	0.99	0.03	0.02
Quality of life (T2)	77.3 (10.4)	73.1 (16.1)	76.8 (9.6)			
MYMOP Profile (T0)	4.0 (0.7)	3.9 (1.2)	3.7 (0.7)	-1.02 (40)	-3.42 (33)	-2.57 (37)
MYMOP Profile (T1)	2.8 (0.9)	3.1 (1.2)	3.7 (0.9)	0.31	0.002	0.01
MYMOP Profile (T2)	3.1 (1.4)	3.3 (1.2)	3.0 (0.8)			

In the verum and sham acupuncture groups T1 is the end of the treatment and T2 is a three-month follow-up after the end of treatment. In the waiting control group T1 is the end of the waiting time and T2 the end of verum acupuncture treatment. Repeated measurement ANOVAs comparing the three groups showed p-values <0.05 for the time x group interaction for all variables except the PHQ stress module. The Table presents the t- and p-values of subsequent contrasts

Results of the ANOVAs using T0 and T1 repeated measurements indicated a significant change over time for all variables. In addition, for all variables except the PHQ stress module the group x time interaction showed a p-value < 0.05. Subsequent t-tests revealed that the verum and sham acupuncture groups showed better outcomes compared to the waiting list group.

In 2005, Fliege et al. [28] had published mean values and standard deviations of various patient groups and a group of healthy persons. The group of n = 334 healthy adults showed a mean PSQ-20 score of 33 (± 17). If we define “clinically significant change” as being within two standard deviations from the mean of the functional or healthy group [38] then a post-treatment mean score < 67 would indicate a clinically significant change for the “dysfunctional” population (i.e. the participants with initially high stress levels). Interestingly, the waiting control group still showed a mean stress level above this cut-off after the waiting period whereas the mean levels of the verum and sham acupuncture groups were below this cut-off, both at the end of treatment and at follow-up. In addition, the mean stress score of the “waiting group” also dropped below this cut-off after having received the verum acupuncture treatment (T2).

**Fig 2:**
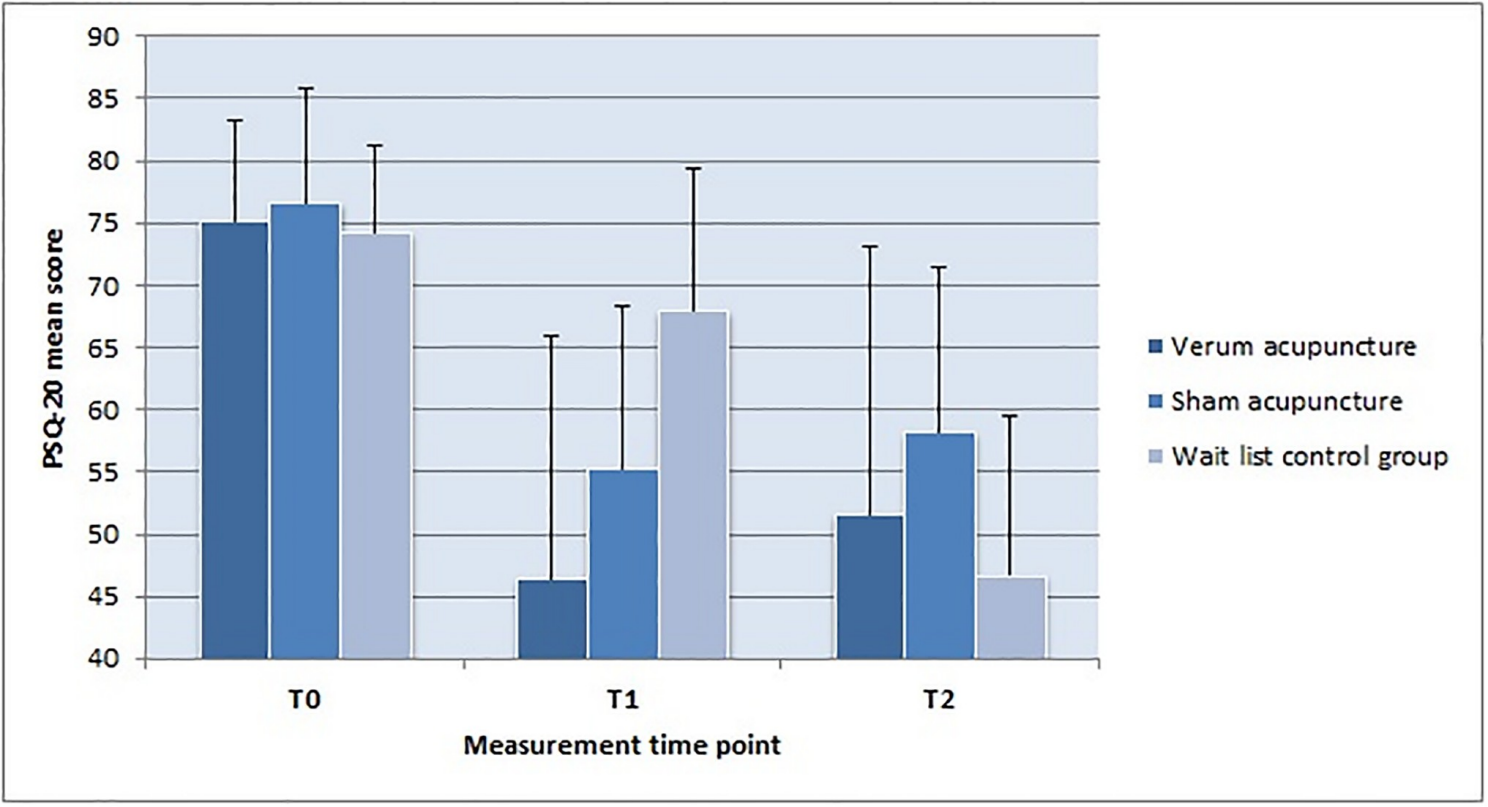
Changes in stress levels over time.

Fig 2 graphically displays the changes in PSQ-20 stress scores of the three groups over the three measurement time points.

Table 3 shows the various effect sizes at the end of treatment or waiting time together with their confidence intervals.

**Table 3:** Change scores and effect sizes for the PSQ-20 plus additional stress-related variables at T1.

	Verum	Sham	Waiting control	Verum vs. Waiting control	Verum vs. sham	Sham vs. waiting control
Change score(T1-T0)	Mean value (STD)	Mean value (STD)	Mean value (STD)	Effect size (95% CI)	Effect size (95% CI)	Effect size (95% CI)
PSQ-20	-30.6 (20.7)	-22.7 (15.8)	-7.0 (12.5)	**-1.39** (-2.11; -0.67)	-0.44 (-1.05; 0.16)	**-1.12** (-1.78; -0.44)
PHQ stress module	-3.2 (2.6)	-3.0 (3.7)	-1.4 (2.1)	**-0.79** (-1.46; -0.12)	-0.06 (-0.66; 0.54)	-0.53 (-1.17; 0.10)
Depression severity	- 6.4 (4.5)	- 6.1 (4.6)	- 1.9 (4.4)	**-1.0** (-1.72;- 0.35)	-0.06 (-0.66; 0.54)	**-0.96** (-1.62; -0.30)
Anxiety severity	-6.7 (4.4)	-4.7 (4.4)	-1.4 (5.9)	**-1.06** (-1.76; -0.36)	- 0.46 (-1.06; 0.15)	**-0.67** (-1.32; -0-02)
Somatic complaints	-3.1 (3.3)	-3.2 (4.2)	0 (2.6)	**-1.05** (-1.73; -0.36)	0.03 (-0.57; 0.63)	**-0.90** (-1.56; -0.24)
Quality of life	13.4 (15.6)	13.4 (15.1)	0.53 (18.3)	**0.78** (0.10; 1.46)	-0.01 (-0.61; 0.61)	**0.80** (0.15; 1.45)
MYMOP Profile Score	-1.34 (1.2)	-0.98 (1.1)	-0.05 (1.1)	**-1.19** (-1.91; -0.47)	-0.32 (-0.94; 0.29)	**-0.86** (-1.52; -0.19)

All of the effect sizes comparing the verum group with the waiting list group at T1 favoured the verum acupuncture (with confidence intervals not including 0). Regarding the stress level measured by the PSQ-20, the effect size was large (-1.39), in favour for the verum group. The comparison between sham treatment and waiting group also resulted in effect sizes favouring the sham intervention–except for the PSQ-stress module. However, the effect sizes were smaller than those in favour for the verum group; effect sizes comparing the verum *versus* the sham group at T1 were not significant.

The effect size for the comparison between verum and sham regarding the PSQ-20 score at T1 was estimated with- 0.44 (95%CI = [-1.05. 0.16]). This effect size could be used for the sample size calculation of a following larger RCT. Assuming a two-side significance level of 0.05 and a power of 80% to prove the efficacy of verum acupuncture compared to sham acupuncture one would have to include n = 166 participants in a larger two-arm RCT. With a loss-to-follow-up rate of about 16% n = 198 participants should be included in a two-arm RCT study.

Table 4 shows the effect sizes of the comparison between the verum and sham treatments at the three-month follow-up (T2).

**Table 4:** Change scores and effect sizes for the PSQ-20 and additional stress-related variables at T2—Comparison between verum and sham group.

	Verum	Sham	Verum vs. sham	t-test for T2-T0 change score verum vs. sham
Change score(T2-T0)	Mean value (STD)	Mean value (STD)	Effect size (95% CI)	t-value (df) p-value
PSQ-20	-25.5 (22.6)	-19.5 (15.7)	-0.31 (-0.93; 0.30)	-0.99 (33) 0.33
PHQ stress module	-2.9 (2.9)	-2.0 (2.8)	-0.34 (-0.95; 0.28)	-1.05 (33) 0.30
Depression severity	-5.6 (4.8)	-4.8 (3.8)	-0.18 (-0.79; 0.44)	-0.55 (39) 0.58
Anxiety severity	-6.6 (4.8)	-4.1 (4.8)	-0.52 (-1.14; 0.1)	-1.63 (39) 0.11
Somatic complaints	-1.8 (3.7)	-2.8 (4.0)	0.28 (-0.33; 0.90)	0.88 (39) 0.39
Quality of life (EQ-5D)	14.5 (15.7)	12.6 (14.2)	0.13 (-0.48; 0.74)	0.40 (39) 0.69
MYMOP Profile Score	-1.1 (1.6)	-0.77 (1.3)	-0.23 (-0.86; 0.39)	-0.01 (38) 0.99

Negative changes in PSQ-20, PHQ stress, and the other variables indicate improvement. In quality of life, positive changes indicate improvement

Effect sizes for comparison with the waiting group at T2 were not computed because at this time point, the waiting list group had already received verum acupuncture treatment.

Apart from somatoform complaints, effect sizes of all variables are in favour of the verum group. However, confidence intervals and t-tests show that these effect sizes were not significant.

### Heterogeneity among study centres

Additional analyses were run to explore the heterogeneity of the two study centres. These analyses included only the verum and the sham group because change scores at T1 and T2 were investigated. Results of the ANOVA regarding PSQ-20 change scores revealed no differences between study centers regarding T1-T0 change scores in stress. However, for the T2-T0 change scores in stress a significant interaction between centre and treatment arms (F_1,37_ = 5.12, p = 0.03) was found. Subsequent t-tests indicated that in one centre (but not in the other), the T2 stress level of the verum group had significantly improved compared to the sham group. Additional centre-specific ANOVAs regarding change scores in depression, anxiety, and stress levels measured by the PHQ stress module did not indicate different results for the two centres.

### Biological parameters

Table 5 shows the mean values of cortisol and cortisone measured in the urine samples. All of the corresponding effect sizes (not shown) had confidence intervals that included the Zero, indicating that the differences between groups would not be significant.

**Table 5:** Mean values of cortisol and cortisone measured in urine samples at baseline, T1, and T2.

(mg / g Kreatinine)	Verum acupuncture (n = 18)	Sham acupuncture (n = 19)	Waiting control group (n = 13)
	Mean value (STD)	Mean value (STD)	Mean value (STD)
Cortisol (T0)	43.2 (23.3)	42.5 (20.3)	33.7 (16.7)
Cortisol (T1)	33.5 (22.9)	37.0 (23.4)	37.7 (29.8)
Cortisol (T2)	37.4 (16.7)	39.3 (22.6)	39.4 (27.4)
Cortisone (T0)	121.9 (53.7)	112.9 (49.6)	111.4 (56.7)
Cortisone (T1)	94.6 (50.9)	105.2 (56.0)	113.7 (56.8)
Cortisone (T2)	96.0 (39.8)	111.5 (48.2)	117.4 (67.9)

## Discussion

This study demonstrates the feasibility of a three-arm randomized controlled trial for adults with increased stress levels by comparing verum with sham acupuncture and a wait list control condition.

The response to the advertisements of the study showed that in general there is a high interest in acupuncture treatment. For our study, the number of eligible participants was restricted due, primarily, to the required high stress level at baseline (PSQ-20 score ≥ 60). Nevertheless, we were able to include n = 70 participants in the course of a recruitment period of 11 months, thereby almost reaching the planned sample size of n = 75. All included participants were willing to be randomized to one of the three study arms.

Adherence and drop-out rates of the study show that the design and treatment protocol were, in fact, feasible and well accepted. The lost to follow-up rate was low (15.7%) and participants showed a high compliance with the treatment protocol.

The proposed outcome measure–the total score of the PSQ-20—appears to be appropriate for measuring the efficacy of the acupuncture intervention. The items of the PSQ-20 represent the subjective perspective of the individuals; they do not list specific worries, but rather ask whether a person feels under pressure because of them (Levenstein, 1993). Results of our study emphasize that the PSQ-20 score is sensitive to change.

The pilot study provides an estimation for the standard deviations and mean values of the secondary outcome measures. These parameters can be used for the sample size calculation of a later larger RCT. According to the estimations from this pilot trial a a following RCT should include n = 198 participants to prove the efficacy of verum compared to sham acupuncture regarding stress reduction.

Mean values in PSQ-20 stress levels (as well as depression and anxiety severity) were high at baseline. Due to the high cut-off requirements for admission to the study, the standard deviation of the PSQ-20 was relatively small at the beginning, but increased over time.

In our pilot study the estimation of effect sizes at T1 showed that both verum and sham acupuncture were superior to the waiting condition in reducing stress levels. In comparison to the waiting list group effect sizes at T1 were consistently higher for all variables in the verum group than in the sham group. For the PHQ stress module, the effect size comparing verum acupuncture to the waiting list condition was significant, whereas the effect size for sham acupuncture was not. In addition, effect sizes when comparing verum and sham acupuncture at T2 were all (apart from one) in favour of the verum acupuncture. These effect sizes were not significant, however, possibly due to the small sample size.

When interpreting the effect sizes of comparing verum to sham acupuncture one must take into account that sham acupuncture is a complex control treatment. It is not comparable to placebo pills as it differs regarding the aspects of physiological response and blinding efficacy [19, 39]. Sham acupuncture can involve non-specific elements such as expectations of the participants, possible relaxation time-out during the sessions, or the therapeutic relationship. However, sham acupuncture can also induce specific effects from the needling; even when the needling points are chosen outside the meridians (non-acupoints) the needle’s insertion can excite local mechanisms of biochemical and biophysical reactions and thus have an impact on the energy system [40]. Small-scale studies that compared verum to sham acupuncture therefore very rarely reach significance [19, 41]. Larger RCTs are therefore required to prove the differences between the verum and sham acupuncture treatment.

Regarding the efficacy of acupuncture in stress reduction, the results of our pilot-study expand upon the evidence of the few previous RCTs that exist. Huang et al. [19] found that in n = 18 persons with increased stress levels the verum acupuncture group significantly improved in the MYMOP profile (pre-post comparison); however, differences in the stress scores did not reach significance due to the very small study sample. In a three-arm RCT that included n = 120 patients suffering from psychological distress Arvidsdotter et al. [20] showed that verum acupuncture and integrative treatment were significantly better than conventional treatment in all outcome variables. Regardless, the study included patients with a psychiatric diagnosis (such as depression and/or anxiety) but did not explicitly measure stress levels. Using the Perceived Stress Scale as outcome measure, Schroeder et al. [42] reported that compared to sham acupuncture, verum acupuncture resulted in a significantly greater reduction in stress. However, of the initially n = 111 included participants with high self-reported stress levels only n = 62 completed the study. Many participants had dropped out because they could not maintain the required treatment schedule. In contrast, in the AkuRest study the treatment protocol of the acupuncture intervention was clearly well accepted; only a few participants dropped out of the intervention and very few acupuncture sessions were cancelled.

The high mean values and low standard deviations in the PSQ-20 scores at baseline reflect the high stress levels of the participants when they entered the study. In the verum group the mean value dropped below 50 at T1 and slightly increased to 51.6 at T2. Similarly, in the wait list group, the PSQ stress mean value was highly decreased between the end of the waiting period (T1) and the end of the verum acupuncture treatment (T2). The same pattern is also apparent in all the other psychosocial–and endocrinologic–variables. In the verum group, there was a strong improvement between baseline and end of treatment, and a slight decline between end of treatment and follow-up. In the wait list group, there was a very small improvement in all variables during the waiting period, followed by a substantial improvement at the end of the verum acupuncture treatment.

The analyses regarding a possible heterogeneity among the two centres showed no differences in PSQ-20 change scores at T1. At T2, only one centre showed significantly improved PSQ-20 scores for the verum group compared to sham. For further variables, no differences between the two study centers were found. However, as the PSQ-20 score would be our preferred primary outcome for a subsequent larger RCT we would recommend to stratify randomization for centres and to include the centre factor in the outcome analysis.

Our pilot study has several limitations. Firstly, the design included a wait list control arm. The advantage of this control condition is greater attractiveness for entering the study. After the waiting period, 84.2% of the participants in the wait list wished to receive verum acupuncture; the motivation to follow the treatment and measurement protocol was therefore high. However, the disadvantage of this control condition is the lack of a control group for a longer follow-up. As a result, we were not able to estimate the effect size of verum acupuncture *versus* no acupuncture treatment after a period of six months. For a larger RCT, it should be re-thought whether or not a pure no-treatment control condition or a longer waiting period would be preferable. Secondly, we see the problems associated with sham acupuncture as a placebo control treatment. However, we believe that with a larger sample size we would be able to prove the efficacy of verum acupuncture in reducing stress levels compared to sham.

All in all, we can conclude from this pilot study that the treatment protocol of the AKuRest study is feasible and well accepted by persons with high stress levels. Estimations of effect sizes show that acupuncture treatment has a positive effect on stress reduction as well as other health outcomes as compared to no treatment. In addition, results indicate that verum acupuncture could, in fact, be more effective than sham acupuncture in reducing stress levels. However, to scientifically prove the efficacy of verum acupuncture as compared to sham acupuncture an RCT with a larger sample size should be conducted.

## Supporting information

S1 Checklist CONSORT 2010 checklist of information to include when reporting a pilot or feasibility randomized trial in a journal or conference abstract. (DOC)

S1 File (PDF)

S2 File (PDF)

S1 Data (XLS)
