# Supplementary material for: Acupuncture in persons with an increased stress level—Results from a randomized-controlled pilot trial
Source: PLoS One. 2020 Jul 23;15(7):e0236004. doi: 10.1371/journal.pone.0236004 (PMC7377446; doi:10.1371/journal.pone.0236004)
Supplement: S1 Checklist — (DOC) [file pone.0236004.s001.doc]

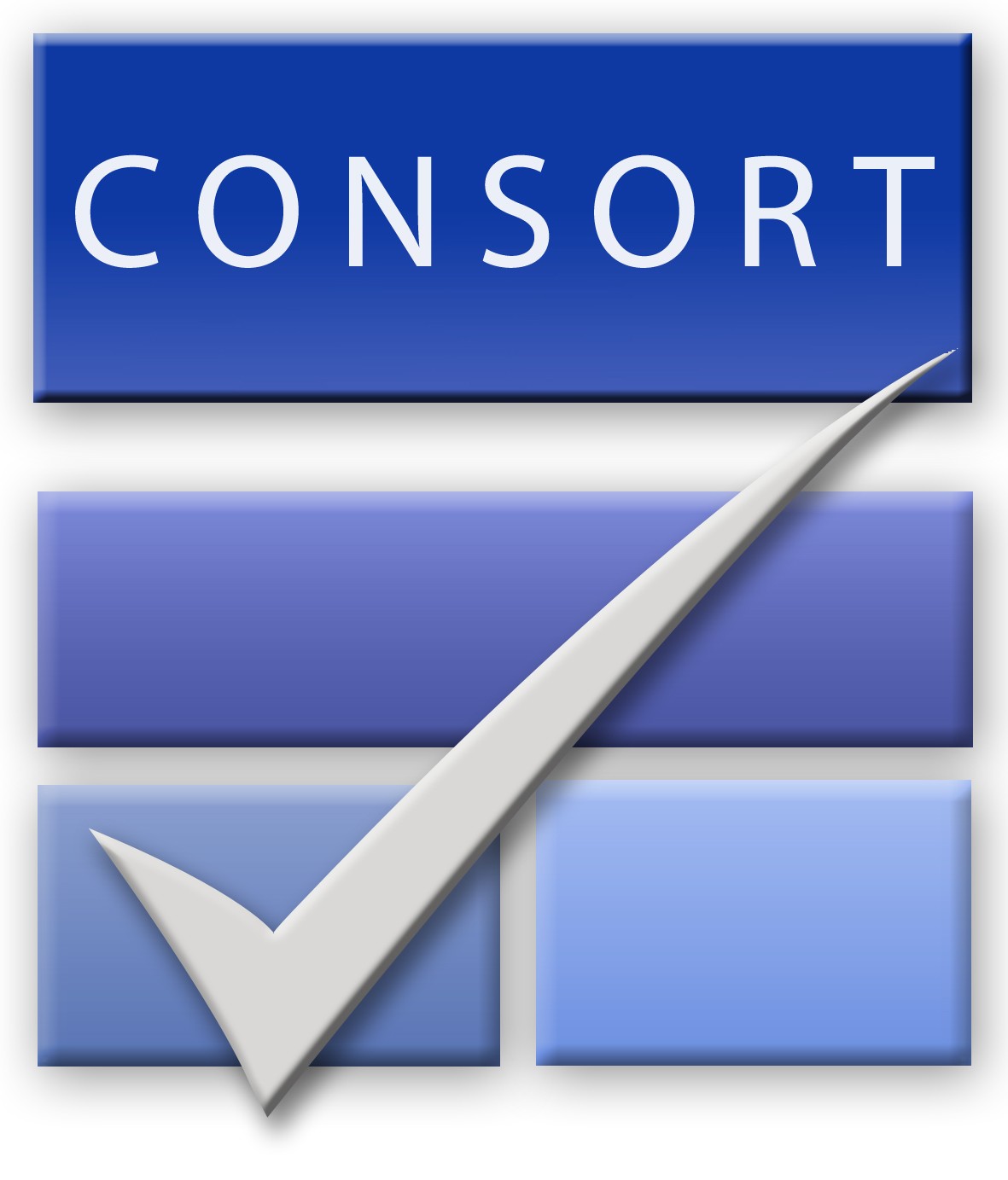
**CONSORT 2010 checklist of information to include when reporting a pilot or feasibility randomized trial in a journal or conference abstract**

| **Item** | **Description** | **Reported on line number** |
| --- | --- | --- |
| Title | Identification of study as randomised pilot or feasibility trial | 1-2 |
| Authors * | Contact details for the corresponding author | 22-28 |
| Trial design | Description of pilot trial design (eg, parallel, cluster) | 37-42, 86-87 |
| Methods |  |  |
| Participants | Eligibility criteria for participants and the settings where the pilot trial was conducted | 94-101 |
| Interventions | Interventions intended for each group | 112-135 |
| Objective | Specific objectives of the pilot trial | 87-90 |
| Outcome | Prespecified assessment or measurement to address the pilot trial objectives** | 138-179 |
| Randomization | How participants were allocated to interventions | 104-109 |
| Blinding (masking) | Whether or not participants, care givers, and those assessing the outcomes were blinded to group assignment | 107-109 |
| Results |  |  |
| Numbers randomized | Number of participants screened and randomised to each group for the pilot trial objectives** | Flow chart |
| Recruitment | Trial status† | 101 |
| Numbers analysed | Number of participants analysed in each group for the pilot objectives** | Flow chart, Table 2 |
| Outcome | Results for the pilot objectives, including any expressions of uncertainty** | 195-280 |
| Harms | Important adverse events or side effects | 226 |
| Conclusions | General interpretation of the results of pilot trial and their implications for the future definitive trial | 364-370 |
| Trial registration | Registration number for pilot trial and name of trial register | 101 |
| Funding | Source of funding for pilot trial | 381-384 |

Citation: Eldridge SM, Chan CL, Campbell MJ, Bond CM, Hopewell S, Thabane L, et al. CONSORT 2010 statement: extension to randomised pilot and feasibility trials. BMJ. 2016;355.

**this item is specific to conference abstracts*

***Space permitting, list all pilot trial objectives and give the results for each. Otherwise, report those that are a priori agreed as the most important to the decision to proceed with the future*

*definitive RCT.*

*†For conference abstracts.*
