# Supplementary material for: Acupuncture in persons with an increased stress level—Results from a randomized-controlled pilot trial
Source: PLoS One. 2020 Jul 23;15(7):e0236004. doi: 10.1371/journal.pone.0236004 (PMC7377446; doi:10.1371/journal.pone.0236004)
Supplement: S1 File — (PDF) [file pone.0236004.s002.pdf]

UniversitätsKlinikum Heidelberg

Studienprotokoll

Version 3.1 vom 20.09.2017

Pilotstudie zur Wirksamkeit von Akupunktur bei Personen mit erhöhtem Stressniveau (AkuReSt)

Studienleitung
Apl. Prof. Dr. Beate Wild
Abteilung für Psychosomatische und Allgemeine Klinische Medizin
Medizinische Universitätsklinik Heidelberg
Im Neuenheimer Feld INF 410,
69120 Heidelberg
Tel.: 06221/56-8663
Fax: 06221/56-5749
E-mail: Beate.Wild@med.uni-heidelberg.de

Kooperationspartner:
Prof. Dr. med. Stefanie Joos
Institut für Allgemeinmedizin und Interprofessionelle Versorgung
Universitätsklinikum Tübingen
Österbergstraße 9, 72074 Tübingen
Tel.: 07071/29 85226; Fax: 07071/29 5896
E-mail: stefanie.joos@med.uni-tuebingen.de

Prof. Dr. med. Yvonne Samstag
Institut für Immunologie
Universitätsklinikum Heidelberg
Im Neuenheimer Feld 305
69120 Heidelberg
Tel.: 06221/56-4039
Yvonne.Samstag@urz.uni-heidelberg.de

Projektleitung / Wissenschaftliche Mitarbeiter:
Dr. med. Judith Brenner
Panoramastr. 27
69257 Wiesenbach

Biometrische Leitung:
Apl. Prof. Dr. Beate Wild
Abteilung für Psychosomatische und Allgemeine Klinische Medizin

Kooperationspartner:
Prof. Dr. med. Roman Huber
Institut für Umweltmedizin und Krankenhaushygiene
Universitätsklinikum Freiburg
Breisacher Straße 115b, 79106 Freiburg
Tel.: 0761/27082010; Fax: 0761/27083230
E-mail: roman.huber@uniklinik-freiburg.de

Sponsor: keiner

Heidelberg, den 20.09.2017

B. Wild

Prof. Beate Wild

Zusammenfassung

Die vorliegende Studie ist eine Pilotstudie für eine nachfolgende, große RCT-Studie zur Überprüfung der Wirksamkeit von Akupunktur bei erhöhtem Stress. Die explorative Studie ist dreiarmig geplant mit einer halbstandardisierten Akupunktur nach Traditioneller Chinesischer Medizin, einer Sham-Akupunktur sowie einer Wartelistengruppe. Vor Beginn und nach Abschluss der Behandlung werden klinische und psycho-neuro-immunologische Parameter sowie die Herzratenvariabilität untersucht, um die Größenordnung der Effekte der Akupunktur einzuschätzen. Darüber hinaus untersucht die Pilotstudie die Machbarkeit des Designs.

Inhaltsverzeichnis

Zusammenfassung 2
1 Einleitung 4
2 Ziele der Studie 4
2.1 Zielkriterien 5
2.2 Hypothesen 5
3 Prüfverfahren 6
4 Studiendesign 6
5 Ein- und Ausschlusskriterien 7
6 Randomisierungsverfahren 7
7 Studienablauf 8
8 Begleittherapie 8
9 Risiken und Sicherheitslabor 8
10 Abbruchkriterien 9
11 Statistisches Design/Datenanalyse 9
12 Rechtliche und Ethische Aspekte 10
13 Versicherung 10
14 Unterschriften zum Protokoll 10
15 References 11
Anhang 12

1 Einleitung

Stress ist in unserem Alltagsleben ein häufig verwendeter Begriff. Dabei wird Stress von den Menschen sehr unterschiedlich wahrgenommen und assoziiert mit Faktoren wie Arbeitsbelastung, Lärm, finanziellen Sorgen und anderem.

Etwas allgemeiner kann man Stress definieren als Bedrohung des inneren Gleichgewichts durch sowohl innere als auch äußere Störreize, sogenannte Stressoren. Hans Selye, der „Vater der Stressforschung“, bezeichnete Stress als die unspezifische Reaktion des Organismus auf jegliche Anforderungen (Selye, 1956)
An sich ist Stress eine normale Reaktion des Körpers und hilft, uns an unterschiedlichste Situationen anzupassen. Sind Menschen einer belastenden Situation ausgesetzt, so reagiert der Körper, in dem er kurzfristig alle Körperfunktionen reduziert, um rasch mit Hilfe von Reflexen auf die Belastung zu reagieren. Wird die Situation erfolgreich bewältigt, tritt eine Erholungsphase ein. Probleme treten auf, wenn die Situation nicht erfolgreich oder angemessen beendet werden kann: Es kommt dann zu einer Phase der Erschöpfung, in der der Körper in Daueralarm ist und geschwächt wird.

Es ist bekannt, dass chronischer Stress ein Risikofaktor oder Verstärker für verschiedenste körperliche Beschwerden bzw. Erkrankungen ist. Chronischer Stress führt nachweislich zu einer Erhöhung des kardiovaskulären Risikos, zu vermehrten muskuloskelettalen Beschwerden und zu psychischen Erkrankungen (z.B. Depression) (Hammen *et al.*, 2009, Rosengren *et al.*, 2004). Darüber hinaus wurde gezeigt, dass eine chronische Stressbelastung zu einer Veränderung der Immunlage führt mit erhöhter Infekthäufigkeit. Diese Entwicklungen sind nicht nur für den einzelnen Patienten bedrohlich, sondern stellen auch auf Public Health - Ebene ein großes Problem dar. Präventive Maßnahmen, die die Stressbelastung verringern und die Folgeerkrankungen vermindern können, sind daher dringend erforderlich.

Im Rahmen der Traditionellen Chinesischen Medizin wird Akupunktur zur Behandlung von stressbedingten Beschwerden eingesetzt. Es gibt erste Studien, die darauf hindeuten, dass Akupunktur bei erhöhtem Stress eine geeignete Behandlung sein könnte (Huang *et al.*, 2011). Bisher fehlen jedoch qualitativ hochwertige randomisiert-kontrollierte (RCT) Studien, die die Wirksamkeit von Akupunktur bei chronischer Stressbelastung belegen könnten.

2 Ziele der Studie

Die geplante Studie (AkuReSt) ist als explorative Pilotstudie konzipiert mit dem Ziel, die Machbarkeit des Designs zu überprüfen sowie Effektgrößen einzuschätzen im Hinblick auf klinische sowie psychoneuroimmunologische und psychophysiologische Parameter zu.

2.1 Zielkriterien

Machbarkeit

Ein Zielkriterium der Pilotstudie ist die Machbarkeit. Als machbar wird die Studie definiert, wenn zumindest 70% der eingeschlossenen Patienten die Studie beenden. Weiterhin wird die Rekrutierungsrate, die Akzeptanz der Randomisierung und die Adhärenz zu der Intervention erfasst.

Fragebögen und psycho-neuro-immunologische Messungen

Ein patientenrelevantes Zielkriterium der Pilotstudie ist die Stressreduktion – gemessen über validierte Fragebögen, den Percieved Stress Questionnaire (PSQ-20) und den Stressfragebogen des PHQ (Fliege *et al.*, 2005, Löwe *et al.*, 2001). Dabei ist der PSQ-20 das geplante Hauptzielkriterium für die nachfolgende RCT-Studie. Aus der Schätzung dieses Endpunktes soll die Fallzahlplanung für eine konfirmatorische Folgestudie abgeleitet werden.

Weitere Zielkriterien, die über Fragebögen erhoben werden, sind körperliche Beschwerden, Depressivität und Generalisierte Angst (PHQ) (Löwe et al., 2001), Lebensqualität (EQ-5D) (Hinz et al., 2014) und das selbsteingeschätzte medizinische Ergebnisprofil (Measure Yourself Medical Outcome Profile (MYMOP) (Hermann et al., 2014).

Zusätzlich zu den Fragebogenmessungen werden psycho-neuro-immunologische und metabolische Untersuchungen durchgeführt. Da bisher kaum etwas bekannt ist über die psychophysiologischen Wirkungen von Akupunktur bei erhöhtem Stress, sollen explorativ verschiedene immunologische und Stress- Parameter als Marker für Gleichgewicht oder Störungen untersucht werden (prä-post Behandlung).

Zusätzlich wird die Herzratenvariabilität der Teilnehmer vor und nach der Behandlung (bzw. Wartezeit) untersucht.

2.2 Hypothesen

Folgende Hypothesen sollen im Rahmen der Studie überprüftwerden:

(1) Das Studiendesign wird gut akzeptiert von den Personen, die den Einschlusskriterien genügen. Die Rate der einwilligenden Personen beträgt über 50%. Darüber hinaus beenden mehr als 70% der eingeschlossenen Personen die Studie.

(2) Explorativ: Die Akupunktur- Intervention führt zu einer klinisch relevanten Reduktion des Stressniveaus (gemessen mit dem PHQ-Stress und dem PSQ).

(3) Explorativ: Akupunktur führt zu einer Veränderung psychoneuroimmunologischer und

psychophysiologischer Parameter.

(4) Verum-Akupunktur zeigt in der Tendenz eine größere Stressreduktion als SHAM-Akupunktur. Signifikante Ergebnisse werden hier erst in einer größeren Studie erwartet.

(5) SHAM-Akupunktur zeigt im Vergleich zur Kontrollgruppe eine Stressreduktion.

3 Prüfverfahren

Im Rahmen der Studie werden personenspezifische Daten (Geschlecht, Alter, Gewicht) erhoben. Die Untersuchung beinhaltet zudem die Selbsteinschätzung des Stressniveaus, der Lebensqualität und der körperlichen Beschwerden (Fragebögen) sowie die Messung der Herzratenvariabilität vor und nach Akupunktur.

Die psycho-neuro-immunologischen und metabolischen Parameter werden im Rahmen von Blutuntersuchungen sowie Urinanalysen erhoben.

4 Studiendesign

Die Studie ist geplant als explorative randomisiert- kontrollierte Studie. Sie hat ein prospektives Design mit drei Messzeitpunkten: T0 (Baseline-Messung), T1 (zum Ende der Behandlung bzw. Wartezeit) und T2 (Follow-up-Erhebung nach 3 Monaten). Verglichen werden eine Interventionsbedingung (Verum-Akupunktur), eine Kontrollbedingung (Sham-Akupunktur) bzgl. des Akupunkturverfahrens und eine Warte-Kontroll-Gruppe (Wartegruppe). Es sollen 3 x 25 = 75 Personen eingeschlossen und auf drei Bedingungen randomisiert werden.

Die Interventionsbedingung besteht aus 10 Sitzungen (von jeweils 20-30 Minuten) Die Akupunkturbehandlung wird an allen drei Standorten von einem/r ausgebildeten Akupunkteur/in (Zusatzbezeichnung Akupunktur der Ärztekammer oder im Besitz der gleichen Anzahl an Ausbildungsstunden) durchgeführt. Der Abstand zwischen den Behandlungen soll personenabhängig je nach Plan- und Durchführbarkeit zwischen 3 Tagen und 7 Tagen betragen.

Für die Akupunktur werden sterile Einmalnadeln mit der Größe 0,25 x 25 (mm) verwendet.

Dabei wird für die Verum-Akupunktur eine halbstandardisierte Auswahl zwischen 3 vorgegebenen und bis zu 4 weiteren individuell wählbaren Akupunkturpunkten angewendet (insgesamt max. 7 Akupunkturpunkte und max. 12 Nadeln). Die vorgegebenen Punkte werden nach vorausgehender Literaturrecherche und Expertise der Akupunkteure vordefiniert und über die gesamten 10 Sitzungen beibehalten. Bei der Akupunktur wird die Auslösung eines De-Qi Gefühls angestrebt. Die individuellen Punkte können je nach führender klinischer Symptomatik von dem/der durchführenden

Akupunkteur/in frei gewählt und im Verlauf der Behandlung verändert werden.

Nach 10-15 Minuten der Akupunkturbehandlung können die Akupunkturnadeln nochmals vom Akupunkteur stimuliert werden.

Die Kontrollbedingung zu der Verum-Akupunktur ist eine SHAM-Akupunktur-Behandlung. Für die Kontroll-Akupunktur werden 4-6 standardisierte Punkte für die Akupunktur ausgewählt (= max. 12 Nadeln), welche nicht auf Akupunkturmeridianen liegen. Diese werden nur oberflächlich ohne Auslösen eines Sog. De-QiGefühls genadelt. Die Kontrollakupunkurpunkte können analog zur Verum-Akupunktur im Verlauf der Behandlung individuell verändert werden. Die Warte-Kontroll-Bedingung besteht aus einer Gruppe, die zunächst über 3 Monate nicht behandelt wird.

5 Ein- und Ausschlusskriterien

Einschlusskriterien:

- Alter ≥ 18 Jahre
- PSQ-Wert ≥ 60

(Der PSQ-20 enthält 20 Items zu wahrgenommenem Stress im Alltag. Diese Items können auf einer Skala von 1 (=fast nie) bis 4 (=meistens) beantwortet werden. Der Gesamtscore des PSQ – der dann mit vorhandenen Normwerten verglichen werden kann – wird so gebildet, dass zunächst die Itemwerte auf 0-3 transformiert werden und dann durch 3 dividiert (lineare Transformation auf den Bereich 0-1). Die Multiplikation mit dem Faktor 100 ergibt dann einen Gesamtwert zwischen 0 und 100. Ein Gesamtwert über 60 gilt als Indikator für hohen Stress (Kocalevent et al., 2007, Levenstein et al., 1993).

Ausschlusskriterien für die Studie sind:

- Akute Suizidalität
- Akute psychiatrische Erkrankung
- Nadelphobie
- kein ausreichendes Verständnis der deutschen Sprache

6 Randomisierungsverfahren

Die Randomisierung wird mit dem Programm „RANDI2“ durchgeführt; stratifiziert wird nach den drei Zentren.

Die Warte-Kontroll-Bedingung besteht aus einer Gruppe, die zunächst über 3 (6?) Monate nicht behandelt wird und dann eine Verum- Akupunkturbehandlung erhält.

7 Studienablauf

Die möglichen Studienteilnehmer werden über eine Pressemeldung auf die Studie aufmerksam gemacht. Sind die Einschlusskriterien zur Studie erfüllt und geben die interessierten Personen ihre Einwilligung, so werden sie in die Studie eingeschlossen. Es erfolgt eine Baseline-Messung (Fragebogen, Speichel und Blutentnahme sowie Uringewinnung) und die Teilnehmer werden randomisiert. Je nach Gruppenzugehörigkeit erhalten sie dann die in Punkt 4 beschriebenen Behandlungen (Verum-Akupunktur, Sham oder Wartekontrollgruppe).

Die zweite Messung (Fragebogen, Speichel- und Blutentnahme, Uringewinnung) erfolgt am Ende der jeweiligen Behandlung bzw. in der Wartekontrollgruppe nach 3 Monaten Wartezeit. Für alle Teilnehmer wird danach noch ein 3-Monats Follow-Up erhoben (Fragebogen, Speichel- und Blutentnahme, Uringewinnung).

Herzratenvariabilität

Die Herzratenvariabilitätsmessung wird am Anfang und am Ende des Behandlungszyklus durchgeführt. Bei den Patienten in den Akupunkturgruppen wird diese 5 Min. vor der Akupunktur, während (ca. 20 Min.) und 5 Min. nach der Akupunktur durchgeführt (insg. ca. 30 Min). Bei der Kontrollgruppe wird eine 30 Min. Messung zu den gleichen Zeitpunkten in Ruhe durchgeführt.

Blutentnahmen und Uringewinnung

Bei den beiden Blutentnahmen erfolgt jeweils Abnahme von ca. 50 ml Blut (drei Monovetten für Serumgewinnung, zwei Monovetten Heparinblut, drei Monovetten EDTA-Blut). Für die Urinanalyse wird, wenn möglich, Morgenurin verwendet. Die Blutentnahme und Uringewinnung dienen u.a. der Bestimmung inflammatorischer Marker (Proteine, RNA) und der Charakterisierung des Redox-Milieus in Blutzellen und Körperflüssigkeiten.

Überschüssiges Material wird nach der Zentrifugation aliquotiert und sofort bei -80º C eingefroren. Ein -80º C Tiefkühlschrank mit Notkühlung steht in der Abteilung zur Verfügung. Überschüssiges Material wird im Rahmen einer Probenbank über zehn Jahre aufbewahrt.

8 Begleittherapie

Entfällt

9 Risiken und Sicherheitslabor

Die Fragebögen bringen keine Risiken für die Patienten mit sich.

Kurzfristig können bei den Blutentnahmen vorübergehende Reizungen oder blaue Flecken auftreten.

Das Risiko einer Komplikation aufgrund der Akupunkturintervention ist auf Basis vorliegender umfangreicher Daten insgesamt als sehr gering einzustufen.

Folgende unerwünschte Wirkungen sind beschrieben worden: Hämatomentwicklung an der Einstichstelle, örtliche Schmerzen während und nach der Akupunktur, lokale Hautinfektionen, vegetative Begleitreaktionen wie Schwitzen, Hitze/Kältegefühle, Müdigkeit. In seltenen Fällen kann es zu vasovagalen Synkopen unter Akupunktur kommen (White et al., 2001, Wu et al., 2015)

Sicherheitslabor entfällt.

10 Abbruchkriterien

Jede Studienteilnehmerin kann zu jedem Zeitpunkt und ohne Nennung von Gründen die Studienteilnahme abbrechen, ohne dass für seine weitere medizinische Versorgung ein Nachteil entsteht.

Abbruchkriterien für die gesamte Studie sind nicht bekannt.

11 Statistisches Design/Datenanalyse

Fallzahlplanung:

Entfällt. Die geplante Anzahl der Teilnehmer entspricht der Fallzahl für eine Pilotstudie. Die Anzahl der Teilnehmer erlaubt eine Subgruppenanalyse getrennt nach Männern und Frauen.

Statistische Auswertung:

Da die Studie eine Pilotstudie ist, entfällt eine konfirmatorische Auswertung. Die Machbarkeit der Studie wird über die Rekrutierungsrate, die Akzeptanz der Behandlung und über die Abbruchrate bzw. Lost-to-follow-up Rate beschrieben.

Die Veränderungen in den weiteren Zielkriterien werden über Effektstärken mit ihren Konfidenzintervallen geschätzt. Deskriptive Analysen von den psycho-neuro-immunologischen und metabolischen Parametern werden durchgeführt. Es werden Differenzwerte mit ihren Konfidenzintervallen berechnet

12 Rechtliche und Ethische Aspekte

Die Untersuchung wird in Übereinstimmung mit der Deklaration von Helsinki in der aktuellen Fassung von 2013 (64th WMA General Assembly, Fortaleza, Brazil, October 2013).

Die Teilnahme der Patienten/Probanden an der Untersuchung ist freiwillig. Die Zustimmung kann jederzeit, ohne Angabe von Gründen und ohne Nachteile für die weitere medizinische Versorgung, zurückgezogen werden.

Die Patienten/Probanden werden vor Studienbeginn schriftlich und mündlich über Wesen und Tragweite der geplanten Untersuchung, insbesondere über den möglichen Nutzen für Ihre Gesundheit und eventuelle Risiken, aufgeklärt. Ihre Zustimmung wird durch Unterschrift auf der Einwilligungserklärung dokumentiert. Bei Rücktritt von der Studie wird bereits gewonnenes (Daten-) Material vernichtet oder beim Patient/ Proband angefragt, ob er mit der Auswertung des Materials einverstanden ist.

Der Untersuchungsplan wird vor Studienbeginn der Ethikkommission der Medizinischen Fakultät Heidelberg zur Begutachtung vorgelegt. Es wird nicht mit dem Einschluss von Patienten/Probanden begonnen, bevor nicht das schriftliche Votum der Ethikkommission vorliegt.

Die Namen der Patienten/Probanden und alle anderen vertraulichen Informationen unterliegen der ärztlichen Schweigepflicht und den Bestimmungen des Bundesdatenschutzgesetzes (BDSG). Eine Weitergabe von Patienten-/ Probandendaten erfolgt ggf. nur in anonymisierter Form.

13 Versicherung

Die Universitätsklinika in Heidelberg, Tübingen und Freiburg sowie deren an der Studie mitwirkenden Mitarbeiter (Studienärzte, sonstiges Personal) sind haftpflichtversichert.

14 Unterschriften zum Protokoll

B. Wild

15 References

Fliege, H., Rose, M., Arck, P., Walter, O. B., Kocalevent, R. D., Weber, C. & Klapp, B. F. (2005). The Perceived Stress Questionnaire (PSQ) reconsidered: validation and reference values from different clinical and healthy adult samples. *Psychosom Med* **67**, 78-88.
Hammen, C., Kim, E. Y., Eberhart, N. K. & Brennan, P. A. (2009). Chronic and acute stress and the prediction of major depression in women. *Depress Anxiety* **26**, 718-23.
Hermann, K., Kraus, K., Herrmann, K. & Joos, S. (2014). A brief patient-reported outcome instrument for primary care: German translation and validation of the Measure Yourself Medical Outcome Profile (MYMOP). *Health Qual Life Outcomes* **12**, 112.
Hinz, A., Kohlmann, T., Stobel-Richter, Y., Zenger, M. & Brahler, E. (2014). The quality of life questionnaire EQ-5D-5L: psychometric properties and normative values for the general German population. *Qual Life Res* **23**, 443-7.
Huang, W., Howie, J., Taylor, A. & Robinson, N. (2011). An investigation into the effectiveness of traditional Chinese acupuncture (TCA) for chronic stress in adults: a randomised controlled pilot study. *Complement Ther Clin Pract* **17**, 16-21.
Kocalevent, R. D., Levenstein, S., Fliege, H., Schmid, G., Hinz, A., Brahler, E. & Klapp, B. F. (2007). Contribution to the construct validity of the Perceived Stress Questionnaire from a population-based survey. *J Psychosom Res* **63**, 71-81.
Levenstein, S., Prantera, C., Varvo, V., Scribano, M. L., Berto, E., Luzi, C. & Andreoli, A. (1993). Development of the Perceived Stress Questionnaire: a new tool for psychosomatic research. *J Psychosom Res* **37**, 19-32.
Löwe, B., Spitzer, C., Zipfel, S. & Herzog, W. (2001). PHQ-D: Gesundheitsfragebogen für Patienten. Pfizer GmbH: Karlsruhe.
Rosengren, A., Hawken, S., Ounpuu, S., Sliwa, K., Zubaid, M., Almahmeed, W. A., Blackett, K. N., Sitthi-amorn, C., Sato, H. & Yusuf, S. (2004). Association of psychosocial risk factors with risk of acute myocardial infarction in 11119 cases and 13648 controls from 52 countries (the INTERHEART study): case-control study. *Lancet* **364**, 953-62.
Selye, H. (1956). *The stress of life*. McGraw-Hill: New York.
White, A., Hayhoe, S., Hart, A. & Ernst, E. (2001). Adverse events following acupuncture: prospective survey of 32 000 consultations with doctors and physiotherapists. *BMJ* **323**, 485-6.
Wu, J., Hu, Y., Zhu, Y., Yin, P., Litscher, G. & Xu, S. (2015). Systematic Review of Adverse Effects: A Further Step towards Modernization of Acupuncture in China. *Evid Based Complement Alternat Med* **2015**, 432467.

Anhang

- Fragebogenset initial
- Fragebogen Follow-up
