# Supplementary material for: Acupuncture in persons with an increased stress level—Results from a randomized-controlled pilot trial
Source: PLoS One. 2020 Jul 23;15(7):e0236004. doi: 10.1371/journal.pone.0236004 (PMC7377446; doi:10.1371/journal.pone.0236004)
Supplement: S2 File — (PDF) [file pone.0236004.s003.pdf]

UniversitätsKlinikum Heidelberg

Study protocol

Version 3.1 of 20.09.2017

Pilot study to investigate the efficacy of acupuncture in persons with an increased stress level (AkuReSt)

Principal Investigator
Apl. Prof. Dr. Beate Wild
Abteilung für Psychosomatische und
Allgemeine Klinische Medizin
Medizinische Universitätsklinik Heidelberg
Im Neuenheimer Feld INF 410,
69120 Heidelberg
Tel.: 06221/56-8663
Fax: 06221/56-5749
E-mail: Beate.Wild@med.uni-heidelberg.de

Cooperation partners:
Prof. Dr. med. Stefanie Joos
Institut für Allgemeinmedizin und
Interprofessionelle Versorgung
Universitätsklinikum Tübingen
Österbergstraße 9, 72074 Tübingen
Tel.: 07071/29 85226; Fax: 07071/29 5896
E-mail: stefanie.joos@med.uni-tuebingen.de

Prof. Dr. med. Yvonne Samstag
Institut für Immunologie
Universitätsklinikum Heidelberg
Im Neuenheimer Feld 305
69120 Heidelberg
Tel.: 06221/56-4039
Yvonne.Samstag@urz.uni-heidelberg.de

Coordinator/research assistant:
Dr. med. Judith Brenner
Panoramastr. 27
69257 Wiesenbach

Biometrician:
Apl. Prof. Dr. Beate Wild
Abteilung für Psychosomatische und
Allgemeine Klinische Medizin

Cooperation partner:
Prof. Dr. med. Roman Huber
Institut für Umweltmedizin und
Krankenhaushygiene
Universitätsklinikum Freiburg
Breisacher Straße 115b, 79106 Freiburg
Tel.: 0761/27082010; Fax: 0761/27083230
E-mail: roman.huber@uniklinik-freiburg.de

Sponsor: none

Heidelberg, 20.09.2017

B. Wild
Prof. Beate Wild

Summary

The following study is a pilot study for a subsequent, large RCT to prove the efficacy of acupuncture in persons with increased stress levels. The explorative study is designed as three-arm study with a partially standardized verum acupuncture treatment according to Traditional Chinese Medicine (TCM), a sham acupuncture treatment, as well as a waiting control group. Before the beginning and after the conclusion of the treatment, clinical and psycho-neuro-immunological parameters, together with heart rate variability are examined in order to estimate the magnitude of the effects of acupuncture. In addition, the pilot study investigates the feasibility of the design itself.

Table of Contents

Summary....................2
1 Introduction....................4
2 Goals of the study....................4
2.1 Outcome parameters....................5
2.2 Hypotheses....................5
3 Assessment procedures....................6
4 Study design....................6
5 Inclusion- and exclusion criteria....................7
6 Randomization procedure....................7
7 Course of the study....................7
8 Concomitant therapy /additional treatment....................8
9 Risks and Safety laboratory....................8
10 Stopping rules....................8
11 Statistical design /data analysis....................9
12 Ethical considerations....................9
13 Insurance....................10
14 Signatures of the protocol....................10
15 References....................10
Appendix....................11

1 Introduction

The term „stress“ is an often-used concept in our daily life. But stress is understood differently by people and is associated with factors of life such as overwork, noise, financial worries, etc.

Generally, one can define stress as a threat of inner equilibrium through internal and external threats, so-called stressors. Hans Seyle, father of stress research, defined stress as the non-specific reaction of an organism to various demands (Selye, 1956). In fact, stress is a normal reaction of the body and helps us to adapt to various situations. When a person is confronted by a demanding situation, the body reacts by temporarily reducing all body functions in order to quickly react to the problem, with the help of our reflexes.

Once the situation is successfully dealt with, a recovery phase emerges. Problems arise when the situation cannot be dealt with successfully or appropriately: a phase of exhaustion then emerges, in which the body is in a constant state of alarm, and is consequently weakened.

It is known that chronic stress is a risk factor or amplifier for various bodily complaints or diseases. Chronic stress demonstrably leads to an increase of cardiovascular risk, to increased musculoskeletal complaints, and mental disorders (e.g. depression). (Hammen *et al.*, 2009, Rosengren *et al.*, 2004). Furthermore, it has been shown that high chronic stress levels lead to a change of the immune system together with an increased frequency of infections. These developments are alarming not only for the individual patient – they also represent a major problem in the area of Public Health. Preventive measures to reduce the stress level and subsequent diseases are therefore urgently needed.

Acupuncture, a part of the Traditional Chinese Medicine (TCM), has long been used as a treatment for stress-related disorders. To date, a few studies have shown that acupuncture may serve as treatment for persons with increased stress (Huang *et al.*, 2011). However, well-designed randomized-controlled (RCT) studies demonstrating the efficacy of acupuncture in chronic stress are still lacking.

2 Goals of the study

The planned study (AkuReSt) is designed as an explorative pilot study with the aim of investigating the feasibility of the design itself as well as to estimate effect sizes in relation to clinical and psycho-neuro-immunological parameters.

2.1 Outcome parameters

Feasibility

An outcome parameter of the pilot study is its feasibility. The study is defined as feasible when at least 70% of the included patients complete the study. In addition, the recruitment rate, the acceptance of the randomization, and the adherence to the intervention will be measured.

Questionnaires and psycho-neuro-immunological measurements

A patient-relevant outcome measure of the pilot study is the stress reduction – measured by validated questionnaires such as the Percieved Stress Questionnaire (PSQ-20) and the stress module of the PHQ (Fliege *et al.*, 2005, Löwe *et al.*, 2001). The PSQ-20 is designed to be the primary outcome of the subsequent large RCT study. The sample size calculation of the subsequent large RCT will be based on the estimates of the PSQ-20 changes of the present pilot study.

Secondary outcome parameters, measured by questionnaires, are somatoform complaints, depression severity, general anxiety severity (PHQ), (Löwe et al., 2001), quality of life (EQ-5D) (Hinz et al., 2014), and the self-assessed medical outcome profile (Measure Yourself Medical Outcome Profile (MYMOP) (Hermann et al., 2014).

In addition to the questionnaire measurements psycho-neuro-immunological assessments are conducted. To date, little is known regarding the psychophysiological effects of acupuncture in increased stress; various immunological stress-parameters will therefore be analyzed as markers for balance or disorders (pre-post treatment).

Further, the heartrate variability of the participants will be measured before and after the treatment (or waiting period).

2.2 Hypotheses

The following hypotheses will be investigated in the framework of the study:

(1) The study design is well accepted by persons who met the inclusion criteria. The number of consenting persons is greater than 50%. In addition, more than 70% of the included persons complete the study;

(2) Explorative: The acupuncture intervention leads to a clinically relevant reduction of the stress level (measured by the PHQ-stress module and the PSQ-20);

(3) Explorative: Acupuncture treatment leads to a change in psycho-neuro-immunological and psychophysiological parameters;

(4) Verum acupuncture shows a tendency for greater stress reduction compared to sham acupuncture. Significant results are to be expected in a larger RCT;

(5) Compared to the control group sham acupuncture shows a reduction in stress levels.

3 Assessment procedures

In the frame of the study person-specific data are measured (sex, age, weight). The assessment also includes self-assessment of the stress level, quality of life, and somatic complaints (questionnaires) as well as the measurement of heart rate variability before and after the acupuncture treatment.

The psycho-neuro-immunological parameters are measured by using blood and urine samples.

4 Study design

The study is planned as an explorative randomized controlled trial. The design is prospective with three measurement time points: T0 (baseline measurement), T1 (at the end of treatment or waiting period), and T2 (follow-up after three months). The intervention condition (verum acupuncture) is compared to an active control condition (sham acupuncture) regarding the acupuncture treatment as well as with a waiting control group. N= 3 x 25= 75 persons should be included and randomized to three conditions.

The intervention treatment consists of 10 acupuncture sessions (each session is from 20-30 minutes in length) and is implemented by a licensed acupuncturist (additional title "acupuncturist" or equal amount of training sessions). Intervals between treatments should vary from 3 to 7 days. For all acupuncture sessions, sterile, single-use filiform acupuncture needles, with a length of 25 mm and a diameter of 0.25 mm each, will be used. For the verum acupuncture a semi-standardized protocol is defined according to literature research and the expertise of the acupuncturists. Three acupuncture points are fixed and maintained throughout the 10 sessions; up to four points will be individually selected by the acupuncturist (maximum 7 acupuncture points and 12 needles). The individual points can be chosen and altered during the course of treatment by the acupuncturist in accordance with the main clinical symptoms. Eliciting a De Qi sensation is the aim of the verum acupuncture treatment; after 10-15 minutes of acupuncture treatment the needles can again be stimulated by the acupuncturist.

The active control condition for the verum acupuncture is a sham acupuncture treatment. For the sham acupuncture, 4-6 standardized points of acupuncture that are not located on acupuncture meridians are chosen (i.e. non-acupuncture points). These points are needled only superficially, that is, without eliciting the so-called De Qi sensation; the control acupuncture points can be changed individually during the course of treatment similar to the verum acupuncture (maximum 12 needles).

From the outset, participants in the waiting control group will receive no acupuncture treatments over the course of three months.

5 Inclusion- and exclusion criteria

Inclusion criteria:

- Age ≥ 18 years
- PSQ-score ≥ 60

(The PSQ-20 includes 20 items regarding self-perceived everyday life stress. These items can be answered on a scale ranging from 1 (“almost never”) to 4 (“most of the time”). The total score of the PSQ – which can be compared with norm values – is calculated by transforming the item scores to 0-3, and then by dividing by 3 (linear transformation to the range of 0-1). Multiplication with the factor 100 results in a total score that ranges between 0 and 100. A total score › 60 is an indicator for high stress (Kocalevent et al., 2007, Levenstein et al., 1993).

Exclusion criteria:

- Acute suicidality
- Acute psychiatric disorder
- Needle phobia
- Insufficient knowledge of the German language

6 Randomization procedure

The randomization will be conducted by using the program „RANDI2“ - to be stratified by the participating centers

Participants in the waiting control group will receive no acupuncture treatments over the course of three months, after which they will be offered a verum acupuncture treatment.

7 Course of the study

Eligible study participants will be informed about the study by advertisements. Interested persons will be included in the study if they meet the inclusion criteria and give their informed consent. A baseline measurement will be done (questionnaires, salivary and blood samples, urine samples); participants are then randomized. According to their randomization group they will receive the treatment, as described above (verum acupuncture, sham acupuncture, or waiting control group).

The second assessment (questionnaires, salivary - and blood samples, urine samples) will be done at the end of treatment or waiting time (three months). For all participants, a three-month follow-up will be conducted (questionnaires, salivary- and blood samples, urine samples).

Heart rate variability

The heart rate variability will be measured at both the beginning and end of the treatment.

With the patients in the acupuncture group, these tests will be implemented 5 minutes before the acupuncture treatment (for approx. 20 minutes) and 5 minutes after the acupuncture treatment. (In total, approx. 30 minutes.) In the control group, heart rate variability will be measured during resting time for approx. 30 minutes using the same timelines.

Blood and urine samples

For both blood samples approx. 50 ml of blood are taken (three monovettes for serum samples, two monovettes heparin blood, three monovettes EDTA-blood). For the urine analyses, morning urine is used (when possible). The blood and urine samples are taken to investigate inflammatory markers (proteins, RND) and to characterize the redox milieu in blood cells and body fluids.

Excess material will be centrifuged; aliquots will be stored immediately at ≤ -80º C. A -80º C refrigerator with emergency cooling is provided by the department. Excess material will be stored in the frame of a biobank over the course of 10 years.

8 Concomitant therapy /additional treatment

Not applicable

9 Risks and Safety laboratory

The questionnaires do not pose a risk for the patients.

Following the blood sampling temporary irritations or bruises can occur.

The risk of a complication due to the acupuncture treatment is estimated – on the basis of comprehensive data - as being very low.

The following adverse effects have been described: Development of a haematoma at the injection side, local pain during and after the acupuncture treatment, local skin infections, vegetative side effects such as sweating, feelings of heat/cold, and fatigue. In rare cases, a vasovagal syncope can occur during acupuncture (White et al., 2001, Wu et al., 2015)

Safety laboratory: not applicable.

10 Stopping rules

A patient may withdraw from the study at any time, at his or her own request, for any reason, specified or unspecified, and without penalty or loss of benefits to which the

patient is otherwise entitled.

Stopping rules for the entire study are unknown.

11 Statistical design /data analysis

Sample size calculation:

Not applicable. The planned number of participants corresponds to the sample size of a pilot study. The number of participants allows for a subgroup analysis, separated for men and women.

Statistical analysis:

The study is a pilot study; therefore, a confirmatory analysis will not be done. The feasibility of the study will be described by reporting the recruitment rate, acceptance of treatment and drop-out rate respectively loss-to-follow-up rate.

Changes in further outcome parameters will be estimated with effect sizes including confidence intervals. Descriptive analyses will be conducted for the immunological and metabolical parameters. Mean differences with their confidence intervals are calculated.

12 Ethical considerations

The study will be conducted in accordance with the Declaration of Helsinki in its current version of 2013 (64th WMA General Assembly, Fortaleza, Brazil, October 2013).

Participation of the patients/participants will be voluntary. A patient may withdraw from the study at any time, at his or her own request, for any reason, specified or unspecified, and without penalty or loss of benefits regarding further medical care.

Before the beginning of the study patients /participants will be informed - in writing and verbally - regarding the nature and consequences of the investigation, particularly regarding the possible benefits or risks for their health. Consent of the participants will be documented with a signature on the consent form. In the case of withdrawal of the study the data material acquired will be completely deleted or the patient /participant will be asked if he/she is in agreement with the analysis of the data material.

The study protocol will be submitted to the ethics commitee of the Medical University Hospital Heidelberg. Recruitment of the patients/participants will not begin before the written approval of the ethics committee.

The names of the patients/participants and all other personal information are subject to medical confidentiality and in accordance with the guidelines of the Federal Data Protection Act (Bundesdatenschutzgesetz, BDSG). Any transmission of participants' data shall be exclusively in anonymized form.

13 Insurance

The University hospitals in Heidelberg, Tübingen, and Freiburg, as well as other study colleagues (study doctors, other personnel) have liability insurance.

14 Signatures of the protocol

B. Wild

15 References

Fliege, H., Rose, M., Arck, P., Walter, O. B., Kocalevent, R. D., Weber, C. & Klapp, B. F. (2005). The Perceived Stress Questionnaire (PSQ) reconsidered: validation and reference values from different clinical and healthy adult samples. *Psychosom Med* **67**, 78-88.
Hammen, C., Kim, E. Y., Eberhart, N. K. & Brennan, P. A. (2009). Chronic and acute stress and the prediction of major depression in women. *Depress Anxiety* **26**, 718-23.
Hermann, K., Kraus, K., Herrmann, K. & Joos, S. (2014). A brief patient-reported outcome instrument for primary care: German translation and validation of the Measure Yourself Medical Outcome Profile (MYMOP). *Health Qual Life Outcomes* **12**, 112.
Hinz, A., Kohlmann, T., Stobel-Richter, Y., Zenger, M. & Brahler, E. (2014). The quality of life questionnaire EQ-5D-5L: psychometric properties and normative values for the general German population. *Qual Life Res* **23**, 443-7.
Huang, W., Howie, J., Taylor, A. & Robinson, N. (2011). An investigation into the effectiveness of traditional Chinese acupuncture (TCA) for chronic stress in adults: a randomised controlled pilot study. *Complement Ther Clin Pract* **17**, 16-21.
Kocalevent, R. D., Levenstein, S., Fliege, H., Schmid, G., Hinz, A., Brahler, E. & Klapp, B. F. (2007). Contribution to the construct validity of the Perceived Stress Questionnaire from a population-based survey. *J Psychosom Res* **63**, 71-81.
Levenstein, S., Prantera, C., Varvo, V., Scribano, M. L., Berto, E., Luzi, C. & Andreoli, A. (1993). Development of the Perceived Stress Questionnaire: a new tool for psychosomatic research. *J Psychosom Res* **37**, 19-32.
Löwe, B., Spitzer, C., Zipfel, S. & Herzog, W. (2001). PHQ-D: Gesundheitsfragebogen für Patienten. Pfizer GmbH: Karlsruhe.
Rosengren, A., Hawken, S., Ounpuu, S., Sliwa, K., Zubaid, M., Almahmeed, W. A., Blackett, K. N., Sitthi-amorn, C., Sato, H. & Yusuf, S. (2004). Association of psychosocial risk factors with risk of acute myocardial infarction in 11119 cases and 13648 controls from 52 countries (the INTERHEART study): case-control study. *Lancet* **364**, 953-62.

Selye, H. (1956). *The stress of life*. McGraw-Hill: New York.
White, A., Hayhoe, S., Hart, A. & Ernst, E. (2001). Adverse events following acupuncture: prospective survey of 32 000 consultations with doctors and physiotherapists. *BMJ* **323**, 485-6.
Wu, J., Hu, Y., Zhu, Y., Yin, P., Litscher, G. & Xu, S. (2015). Systematic Review of Adverse Effects: A Further Step towards Modernization of Acupuncture in China. *Evid Based Complement Alternat Med* **2015**, 432467.

Appendix

- Questionnaires Initial
- Questionnaires Follow-up
